# Exploring patient perspectives on EQ-5D-5L data visualization within an individualized decision aid for total knee arthroplasty (TKA) in Alberta, Canada

**DOI:** 10.1186/s12891-024-07304-5

**Published:** 2024-02-29

**Authors:** Jeffrey A. Johnson, Ademola Itiola, Shakib Rahman, Christopher Smith, Allison Soprovich, Lisa A. Wozniak, Deborah A. Marshall

**Affiliations:** 1 School of Public Health, University of Alberta, 2-040 Li Ka Shing Centre for Health Research Innovation, Edmonton, AB T6G 2E1 Canada; 2 Cumming School of Medicine, Health Sciences Centre, University of Calgary, 3330 Hospital Drive NW, Foothills campus, Calgary, AB T2N 4N1 Canada; 3 Alberta Bone and Joint Health Institute, 400 Crowfoot Crescent NW Suite 316, Calgary, AB T3G 5H6 Canada

**Keywords:** PROMs, EQ-5D-5L, Patient decision aid, Data visualization, Patient perspectives

## Abstract

**Background:**

Decision aids can help patients set realistic expectations. In this study, we explored alternative presentations to visualise patient-reported outcomes (EQ-5D-5L) data within an online, *individualized* patient decision aid for total knee arthroplasty (TKA) that, in part, generates individualized comparisons based on age, sex and body mass index, to enhance usability prior to implementation into routine clinical practice.

**Methods:**

We used data visualization techniques to modify the presentation of EQ-5D-5L outcomes data within the decision aid. The EQ-5D-5L data was divided into two parts allowing patients to compare themselves to similar individuals (1) pre-surgery and (2) 1-year post-surgery. We created 2 versions for each part and sought patient feedback on comprehension, usefulness, and visual appeal. Patients from an urban orthopedic clinic were recruited and their ratings and comments were recorded using a researcher-administered checklist. Data were managed using Microsoft Excel, R version 3.6.1 and ATLAS.ti V8 and analyzed using descriptive statistics and directed content analysis.

**Results:**

A total of 24 and 25 patients participated in Parts 1 and 2, respectively. Overall, there was a slight preference for Version 1 in Part 1 (58.3%) and Version 2 in Part 2 (64%). Most participants demonstrated adequate comprehension for all versions (range 50–72%) and commented that the instructions were clear. While 50–60% of participants rated the content as useful, including knowing the possible outcomes of surgery, some participants found the information interesting only, were unsure how to use the information, or did not find it useful because they had already decided on a treatment. Participants rated visual appeal for all versions favorably but suggested improvements for readability, mainly larger font and image sizes and enhanced contrast between elements.

**Conclusions:**

Based on the results, we will produce an enhanced presentation of EQ-5D-5L data within the decision aid. These improvements, along with further usability testing of the entire decision aid, will be made before implementation of the decision aid in routine clinical practice. Our results on patients’ perspectives on the presentation of EQ-5D-5L data to support decision making for TKA treatments contributes to the knowledge on EQ-5D-5L applications within healthcare systems for clinical care.

## Background

Routinely collected patient-reported outcome measures (PROMs) data can be used at *micro* (patient/clinician), *meso* (organization) and *macro* (health system) levels for various purposes [1]. The Alberta Bone and Joint Health Institute (ABJHI) (www.albertaboneandjoint.com), a not-for-profit organization working to improve outcomes for people with musculoskeletal conditions, have collected PROMs (e.g., EQ-5D-5L) on all hip and knee surgery patients in Alberta since 2004 [2, 3] for quality improvement, including variations by region/clinic.

Recently, ABJHI integrated historic population-level EQ-5D-5L dimension-level data (hereby referred to as EQ-5D-5L data) for an online *individualized* patient decision aid for total knee arthroplasty (TKA), in part, to generate individualized comparisons based on age, sex and body mass index (BMI). This allows patients to consider individuals with similar characteristics in each EQ-5D-5L dimension (1) pre-surgery and (2) 1-year post-surgery [4]. Evidence from a pragmatic randomized control trial showed that patients who completed this decision aid had twice the odds of making quality decisions about TKA (OR = 2.08, 95% CI: 1.08 to 4.02) and fewer underwent surgery (71% vs. 83%), suggesting that patients may have tried non-surgical therapies or adjusted their expectations of surgery [5, 6]. With these positive results, ABJHI plans to implement the decision aid into routine clinical practice for people considering TKA. Currently, ABJHI only collects outcomes for patients who undergo surgery [3]. As part of routine implementation, ABJHI will expand PROMs collection to assess outcomes for patients who do not have surgery to enhance the decision aid through iterative improvements.

While the individualized TKA decision aid was developed using International Patient Decision Aid Standards [4, 7] and underwent usability testing, we seized the opportunity to enhance visualization of individualized EQ-5D-5L data prior to implementation in routine practice. The use of visuals can convey information, such as scores, directionality and meaning, more intuitively using intentional color, bolding and symbols, enhancing accurate interpretation of data [8]. Using data visualization recommendations and techniques to present PROMs data can increase patient understanding and interpretation of the data and, thus, improve decision quality and care [8–10]. Examples of data visualization recommendations for PROMs include the positioning and labelling of scores to enhance communication and interpretation [10]. Furthermore, patient-endorsed visualizations of PROMs data can potentially promote patient-centred care and shared decision-making [9]. The objective of this study is to elicit patient feedback on presentation of EQ-5D-5L data (1) pre-surgery and (2) 1-year post-surgery in the areas of comprehension, usefulness, and visual appeal for patients.

## Methods

**Fig. 1:**
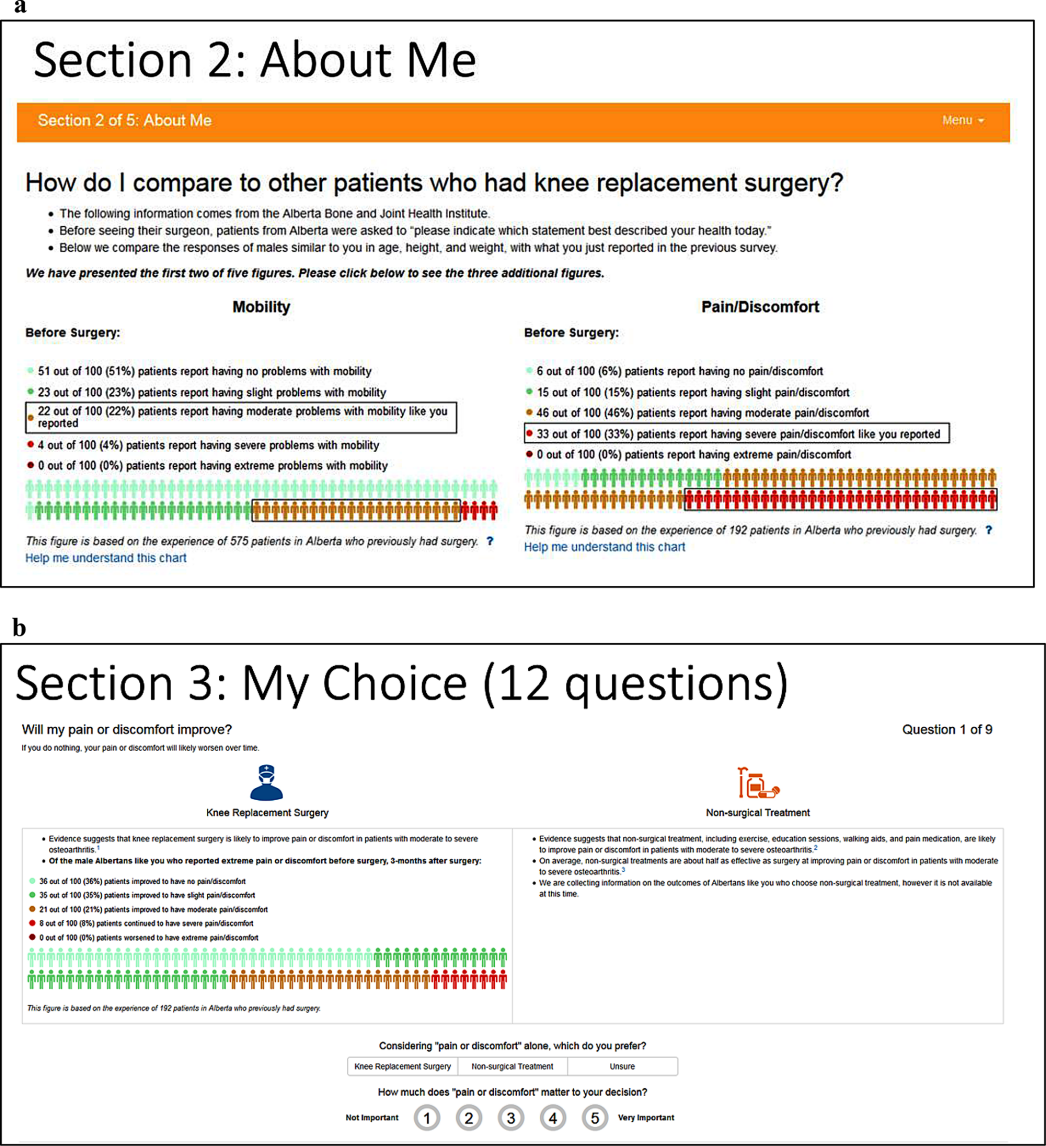
(**a**) EQ-5D-5L data pre-surgery - Original. (**b**) EQ-5D-5L data 1-year post-surgery - Original

**Fig. 2:**
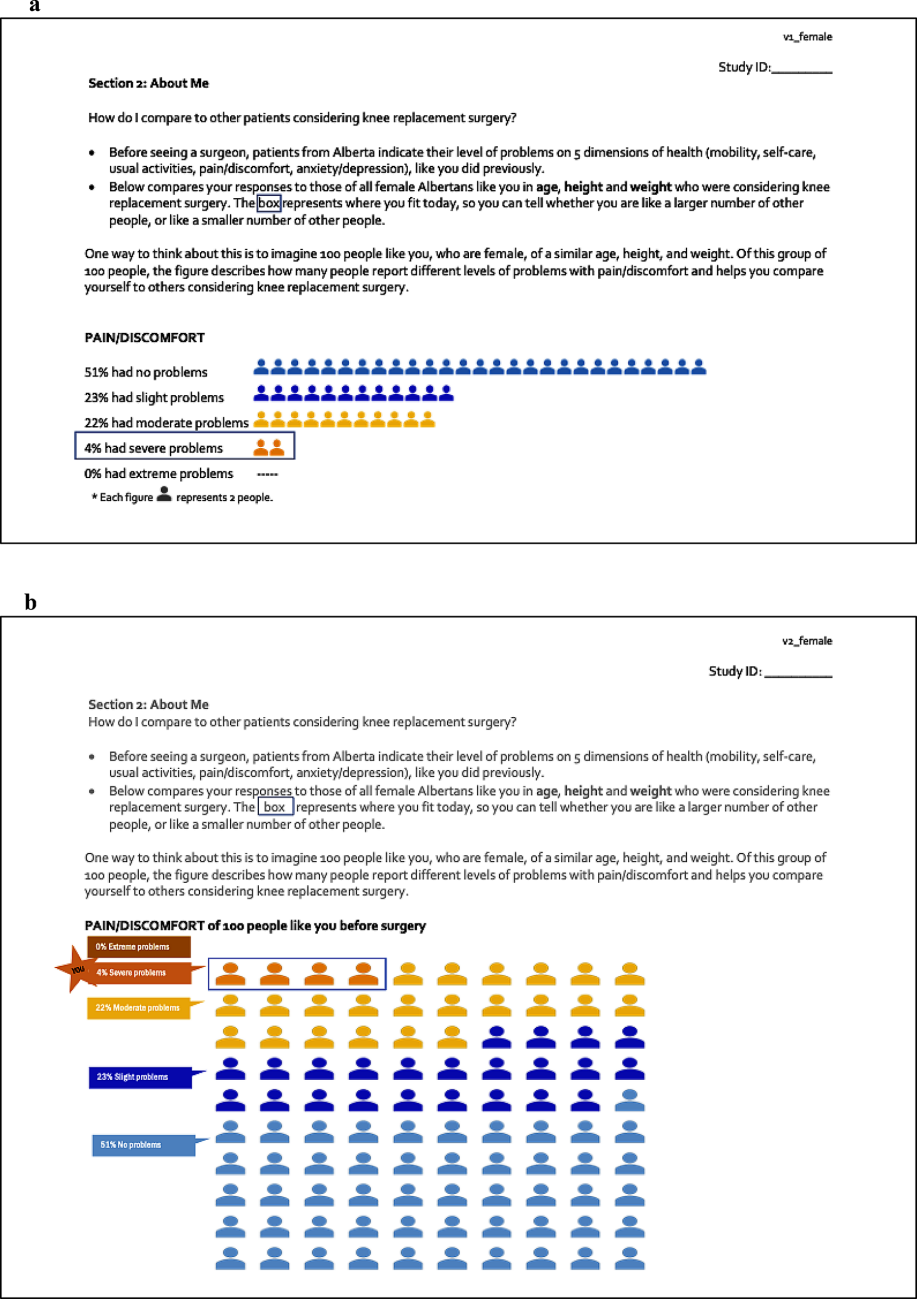
(**a**) EQ-5D-5L data pre-surgery - Part 1, Version 1. (**b**) EQ-5D-5L data pre-surgery - Part 1, Version 2

We solicited patient feedback on different visual presentations of the EQ-5D-5L data within the decision aid for both parts (i.e., pre-surgery and 1-year post-surgery), including instructions to interpret and use the data, through a researcher-administered checklist with closed- and open-ended questions. Prior to collecting feedback, we made purposeful changes to the original instructions and graphics for the EQ-5D-5L data (Fig. 1a/b) in both parts (Table 1; column a). We created prototypes based on recommendations for data visualization [9–17] using the Pain/Discomfort dimension of the EQ-5D-5L: two versions for pre-surgery data (Part 1) and two versions for 1-year post-surgery data (Part 2). We replaced levels (i.e., levels 1) with descriptive labels (i.e., no problems) [10]. For pre-surgery data (Part 1), we replaced male icons with gender-neutral icons based on principles of inclusivity [12, 18]. For 1-year post-surgery data (Part 2), we chose the Sankey Diagram to demonstrate changes over time [19, 20]. Our team underwent three rounds of feedback and used consensus to finalize the prototypes for each part (Fig. 2a/b and 3a/b).

**Table 1:** Summary of modifications & rationale of changes from original to final versions

Colum A: Original Version		Column B: Revised Version 1	Column C: Revised Version 2
**Comprehension**			
Both Parts	Redundant text	Simplified text by removing redundant messaging and/or unnecessary words	
	Repetition of words	Repetitive words removed to be concise	
	Used written numeracy (e.g. 51 out of 100) and proportions (e.g. 51%)	Used proportions (e.g. 51%) only for simplification	
	Use of Jargon/ Scientific / Academic language	Used “plain language”	
	Inconsistent use of terms	Consistency and terms matched EQ-5D-5L scale	
	Long sentence structure	Simple sentence structure used	
	Limited instructions. Part one instructions on how to understand the figure were hidden. Patients needed to click on the link labelled, “? Help me understand this chart” to reveal the information on interpretation. Part two had limited instructions on how to interpret the visualized change in levels or categories of problems before and after surgery.	Part one instructions on how to understand the figure was placed in the narrative above the figure. Part two added instructions on how to interpret the visualized change in levels or categories of problems before and after surgery.	
Part one	Icons were shown separate from the text that described the levels or categories of problems. Two boxes were used: One box was used in the text/narrative and a second box was used in figure. No instructions on how to interpret the information in the box.	Icons are linked to the text describing levels or categories of problems for easy understanding. One box used around the icons and text. Included instructions on how to interpret the information in the box.	
	Two lines of icon used, making it difficult to discern count and levels or categories of problems (i.e. proportions) spread over 2 lines in the figure.	One line of icons with each icon representing 2 people to better visualize proportions. Included footnote information on how to interpret proportions: * Each figure [icon] represents 2 people.	Used a waffle chart where each icon represents 1 person to better visualize proportions. No additional information on how to interpret proportions was included.
Part two	Use of icons (i.e. cross-sectional) made it difficult to discern change in levels or categories of problems before and after surgery; implies a static categorization.	Removed icons. Used Sankey Chart (i.e. longitudinal) to better represent change in levels or categories of problems before and after surgery.	Removed icons. Used Sankey Chart (i.e. longitudinal) and stacked bars to better represent change in levels or categories of problems before and after surgery.
		Used a greater slope of curves indicating magnitude of change.	Used less slope of curves indicating magnitude of change.
		Placement of labels. -External to slope of change -Percentages listed first followed by the label	Placement of labels. -Embedded into the stacked bars -Percentages listed second after label
	There was no recall of a patient’s EQ-5D-5L data on the level or category of problems before surgery. This limited people’s ability to assess their potential probability of change from before to after surgery.	We used narrative recall of EQ-5D-5L data on the level or category of problems before surgery.	We used visual recall of EQ-5D-5L level or category of problems before surgery.
**Usefulness of content**			
Part one	Used internationally accepted ‘Male’ icon for figure.	Used gender-neutral icons (i.e. head and shoulders for body)	
**Visual appeal**			
Both parts	Difficult to discern change of level or category with current colors of icons	Intentional use of color. We used shades of blue to represent less problems (i.e. neutral/calming type color) and yellow, orange, and dark orange to represent more problems (i.e. ‘warning’ type colors).	
	Original default colors	Used ABJHI Branding (i.e. color palette)	

In collaboration with clinic staff to ensure noninterference with clinic flow, participants were recruited from a high-volume urban hip and knee clinic after their surgical screening appointment from April 6 to May 11, 2023. A sample of 10–20 participants has been shown to identify 80–95% of usability issues [21]. In addition, we recruited until we obtained a diverse sample by age, sex, and ethnic background. Eligible participants were ≥ 18 years old; diagnosed with knee osteoarthritis (OA), and referred to the clinic as possible candidate for surgery; and could read, speak, and understand English and provide informed consent. Anyone with soft tissue knee injuries, rheumatoid arthritis, or other non-OA knee conditions was excluded. Patients were approached by a researcher after their physician appointment to inform them of the study and obtain written consent.

Participants completed a demographics form (age, sex, gender identity, and ethnic background) and reviewed the EQ-5D-5L prototypes on the pain/discomfort dimension for female or male based on self-identification. Prototypes represented a hypothetical patient with severe problems (level 4), not the participants’ actual EQ-5D-5L scores. For feasibility, prototypes were presented to participants separate from the entire decision aid and as paper copies, rather than online. Participants compared and contrasted 2 prototype versions for Parts 1 and 2 while research assistants recorded participants’ ratings and comments using a checklist that we developed in the areas of comprehension (9 items), usefulness (5 items), and visual appeal (4 items) for the purpose of this study. There were no validated surveys or checklists in the existing literature appropriate to answer our research question. Therefore, we developed questions using the key concepts in data visualization of comprehension (i.e. understandability of the message), usefulness of the message, and visual appeal in the presentation of the message. We piloted the data collection process, including checklist, and made improvements to enhance quality of the data gathered. First, we conducted mock administration of the process and checklist internally with research staff not directly involved in the study. Second, we piloted the process and checklist in clinic with 10 patients. We held team meetings to discuss the data process and improvements were made based on consensus in both stages.

Closed-ended data were managed using Microsoft Excel, R version 3.6.1 and analyzed using descriptive statistics. We used mean and standard deviation (SD) to summarize age, while frequencies and percentages were used to summarize categorical outcomes. Participants’ ratings (ranging from 1 low/poor to 5 high/positive) of the versions were compared using Wilcoxon signed rank test and McNemar’s test, respectively [22]. Open-ended data was managed using ATLAS.ti Version 8 [23] and analyzed using directed content analysis [24] with comments coded by comprehension (i.e., ability and ease in understanding the information), usefulness of the content, and visual appeal (i.e., aesthetics and readability).

## Results

Participant demographics are reported in Table 2. Overall, there was a slight preference for Version 1 in Part 1 (58.3%) and Version 2 in Part 2 (64%). No statistical differences between versions were noted in any variable or category for either part (Table 3). Below, we report results by Part 1 (pre-surgery) and Part 2 (1-year post-surgery) for comprehension and usefulness. Visual appeal is reported across both parts due to commonality of the results. Due to the purpose of this study and the trademark of the instrument, feedback on the EQ-5D-5L descriptive labels was not reported. Illustrative comments are labeled with study ID (e.g., 01), sex (i.e., “F” for Female and “M” for Male), and ten-year age category (e.g., 50–59).

**Table 2:** Participant demographic information

	Part 1: EQ-5D-5L pre-surgery (n = 24)	Part 2: EQ-5D-5L1 year post-surgery (n = 25)
Age mean(SD) years	67.7 (8.8)	69.0 (7.9)
Sex n(%)		
Female	15 (62.5)	11 (44)
Male	9 (37.5)	14 (56)
Gender n(%)		
Female	15 (62.5)	11 (44)
Male	9 (37.5)	14 (56)
Ethnicity n(%)		
Aboriginal	1 (4.2)	1 (4)
Chinese	1 (4.2)	1 (4)
Fillipino	1 (4.2)	2 (8)
South Asian	3 (12.5)	1 (4)
West Asian	0	1 (4)
White	18 (75.0)	19 (76)

**Table 3:** Participants rating of sample materials

Comprehension	Part 1 (n=24)			Part 2 (n=25)		
	Version 1 n(%)	Version 2 n(%)	p value	Version 1 n(%)	Version 2 n(%)	p value
Understanding of message						
1 (I don’t understand this message)	1 (4.2)	1 (4.2)	0.5863*	0	0	0.5393*
2	4 (16.7)	3 (12.5)		1 (4)	1 (4)	
3	6 (25.0)	8 (33.3)		7 (28)	5 (20)	
4	4 (16.7)	6 (25.0)		9 (36)	9 (36)	
5 (I completely understand this message)	9 (37.5)	6 (25.0)		8 (32)	9 (36)	
No response				0	1(4)	
Clarity of instructions**						
Yes	23 (95.8)			25 (100)	24 (96)	
No	0			0	0	
Don’t know	1 (4.2)			0	0	
No response				0	1 (4)	
Helps understand how patient compares						
Yes	23 (95.8)	21 (87.5)		14 (56)	23 (92)	
No	0	0		8 (32)	1 (4)	
Don’t know	1 (4.2)	2 (8.3)		2 (8)	1 (4)	
No response	0	1 (4.2)		1 (4)	0	
**Usefulness**						
Usefulness of information						
1 (Not useful at all)	1 (4.2)	2 (8.3)	0.7011*	1 (4)	0	0.4856*
2	1 (4.2)	3 (12.5)		3 (12)	3 (12)	
3	10 (41.7)	7 (29.2)		6 (24)	6 (24)	
4	7 (29.2)	6 (25.0)		10 (40)	8 (32)	
5 (Very useful)	5 (20.8)	6 (25.0)		5 (20)	8 (32)	
**Visual Appeal**						
Visual appeal						
1 (Not visually appealing)	0	5 (20.8)	0.2906*	0	0	0.9226*
2	5 (20.8)	2 (8.3)		3 (12)	4 (16)	
3	7 (29.2)	7 (29.2)		5 (20)	3 (12)	
4	7 (29.2)	7 (29.2)		9 (36)	11 (44)	
5 (Very visually appealing)	5 (20.8)	3 (12.5)		8 (32)	7 (28)	
**Preferred material**						
Overall	14 (58.3)	10 (41.7)	0.5403***	9 (36)	16 (64)	0.23201***

*Wilcoxon signed rank test with continuity correction

**Instructions were identical between versions for Part 1

***McNemar’s Chi-squared test with continuity correction

### Part 1: EQ-5D-5L data pre-surgery (Fig. 2a/b)

There were 24 participants for Part 1. The mean age was 67.7 (± 8.8) years, 62.5% were female, and 75% reported their ethnicity as white (Table 2).

#### Comprehension

Almost all participants (95.8%) indicated the instructions were clear; of note, the instructions were the same in both versions (Table 3). There were mixed opinions about the simplicity of the instructions with a few participants saying the instructions were *“clear and easy to follow”* (03 F 50–59) or used “*simple language”* (19 F 70–79), while other participants wanted “*simpler terms*” (17 M 80+) or clearer wording (16 M 70–79).

Approximately half the participants rated the materials 4 or 5 (I completely understand this message) for both versions (54.2% for Version 1; 50% for Version 2). For both versions, only 1 participant rated comprehension as 1 (I don’t understand the message). Overall, participants said for Version 1, “*The message is clear*” (01 F 70–79) or “*It’s easier to understand and comprehend*” (11 F 50–59), whereas Version 2 was “*not very user friendly and takes some time to wrap your head around*” (12 F 60–69) or “*too difficult to understand*” (16 M 70–79).

Regardless of version, most participants agreed that the information helped them understand how they compared to others (95.8% stated “yes” this information helps you understand how you compare to other people like you for Version 1, and 87.5% for Version 2) (Table 3). Additionally, most participants recognized the EQ-5D-5L information was for comparison purposes to other people like them: “*This chart [in Version 1] shows how I compare to 100 people like me. I really like how you are comparing to other people that are similar in height and weight to me*” (01 F 70–79). In addition, some patients reported that the box (in both versions) and/or star icon (in Version 2) helped direct their attention to where they would fit in comparison to others.

Participants preferred the icon array presentation in Version 1 (Fig. 2a), with separate rows of icons by level, making it “*easier to understand with the groups on one line each*” (12 F 60–69). The waffle or grid display in Version 2 (Fig. 2b) was more difficult for participants to recognize that no people had ‘extreme problems’ (06 M 60–69) or had too many icons (13 M 60–69; 19 F 70–79). However, a few participants said that Version 2 was “*More representative of a community*” (04 M 70–79), a “*Good sample*” (17 M 80+), or “*a better display of the answers from other people you asked*” (24 F 60–69).

There were varied opinions on the number of people each icon represented, representing approximately 2 people in Version 1 and 1 person in Version 2. In Version 1, some participants understood that “*you need to multiply the number of people by 2*” (03 F 50–59) or “*[It] doesn’t matter about 100 people, the percentages are clear enough for me*” (11 F 50–59). However, for Version 2, a few participants reported that 100 icons made it “*easier to calculate percent from a whole group instead of needing to do extra math*” (04 M 70–79).

#### Usefulness

Half the participants (50%) rated content 4 or 5 (very useful) for both versions. One participant rated the content as 1 (not useful at all), versus 2 participants for Version 2 (Table 3). Regardless of version, many patients did not think the EQ-5D-5L pre-surgery information would be useful or were unsure how to use this information in treatment decision: *“What do I do with this information?”* (13 M 60–69). Some participants remarked on the “*lack of rationale*” (16 M 70–79) for comparing to others and questioned its usefulness: *“I will make the decision based on my own experiences”* (08 F 60–69) or “*Not too sure [this is useful], because I wouldn’t be concerned with how I compare to others*” (14 M 50–59). A few participants said the EQ-5D-5L pre-surgery information was not useful to them because they had already decided on treatment with their doctor, *“No, my doctor and I talked about it and I know I need surgery*” (22 F 60–69).

When asked what the most important information was to them, the majority of participants said, “*How I compare to other people like me*” (07 F 70–79). They said this was “*interesting*” information (08 F 60–69; 15 F 50–59), “*nice to know*” (05 F 70–79), or comforting: *“Some comfort in knowing am in the 22% -23% of the population”* (20 M 80+), implying limited usefulness and without explicitly explaining why the information was important to them or how they would use the information.

Several participants implied that the EQ-5D-5L pre-surgery information might be used to assess their condition and to triage or prioritize one treatment option (e.g., surgery) particularly when they felt it showed the severity of their problems, “*And hopefully that means I can get in to have my surgery sooner*” (03 F 50–59). This perception of severity and/or prioritization for surgery, may or may not be true, depending on individual cases. Regardless, assessment of severity or eligibility for surgery is not the purpose of the EQ-5D-5L pre-surgery information.

### Part 2: EQ-5D-5L data 1-year post-surgery (Fig. 3a/b)

There were 25 participants for Part 2. The mean age was 69 (± 7.9) years, 44% were female and 76% reported white as their ethnicity (Table 2).

**Fig. 3:**
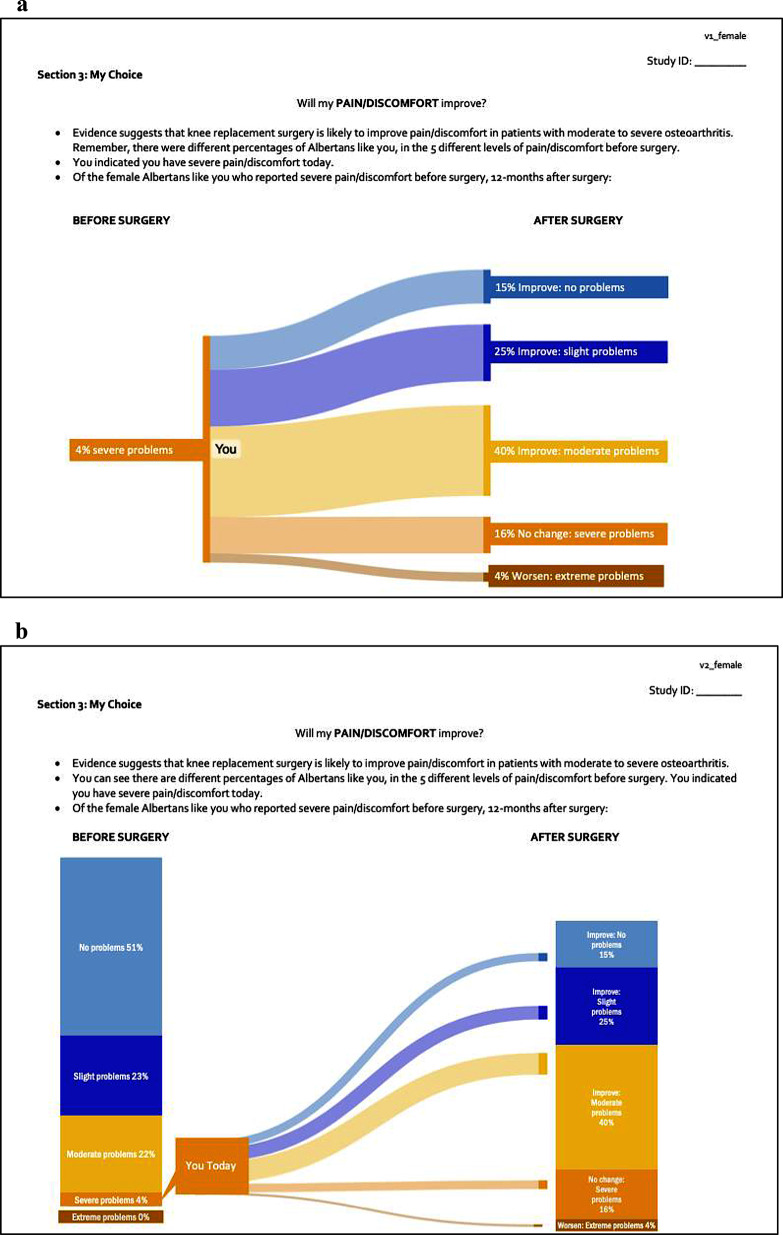
(**a**) EQ-5D-5L data 1-year post-surgery - Part 2, Version 1. (**b**) EQ-5D-5L data 1-year post-surgery - Part 2, Version 2

#### Comprehension

All participants were clear about the instructions provided in both versions, and almost all (95.8%) agreed that Version 2 helped them understand how they compared to others after surgery. Most participants gave an overall rating of either 4 or 5 (I completely understand this message) for both versions (68% for Version 1, 72% for Version 2) (Table 3).

In support of the ratings, participants generally reported the message “*was very clear*” (16 F 70–79) or “*nothing is confusing*” (22 M 60–69) for both versions. Overall, participants demonstrated understanding the information presented in both versions: “*How people like me do after surgery. There is a range of options where some people do very well and have no pain and some stay the same or get worse [in Version 1]*” (10 M 60–69). Of note, some participants recognized more complete information provided in Version 2 by explicitly referencing ‘before surgery’, and preferred this additional information: “*[Version 2] is better because I can see the entire group, the whole 100%, and then follow how I do*” (08 F 60–69). Regardless, some participants found the additional information in Version 2 unnecessary and/or distracting: “*I don’t think I need the information about how I am right now so it’s too busy to look at*” (11 M 70–79). A few participants recommended providing the ‘before’ and ‘after’ surgery information separately: “*This is better to see the whole group before surgery, so I can compare to similar people and then I can see how they do after surgery. It might almost be best to show this one first and then the other one next if you can do that, since both are helpful*” (22 M 60–69).

To improve comprehension, 1 participant recommended additional instructions and changing the headings to be more descriptive: “*Instruct the person to find your discomfort level under the before surgery heading and possible results. Change the language of the headings. Replace ‘Before Surgery’ with your current level of knee problems. And the after surgery with something like expected results or possible outcomes*” (08 F 60–69). Furthermore, some participants recommended removing the label ‘You’ from both versions “*since I know how I’m doing today*” (08 F 60–69) or because it was misleading as “*The visual is such that ‘You’ are in the 40% improvement [Version1]*” (02 M 50–59), making it appear like there was only one possible outcome of surgery, rather than a variety of outcomes.

#### Usefulness

Approximately 60% of participants rated the usefulness of the content as either 4 or 5 for both versions (60% and 64%) (Table 3). For both versions, some participants noted it was “*great information to have*” (18 F 60–69) or “*valuable information to understand*” (04 M 80+). When asked if anything was missing from the materials, most patients said “*nothing*” regardless of version. Most participants found it useful to know the possible outcomes of surgery, including assessing risks and benefits and/or setting realistic expectations:

*It will help me balance between the risk of the surgery and the benefits of it”* (21 M 60–69).

*Some people are worse or the same after the surgery*” (09 F 70–79).

However, similar to feedback on Part 1, some participants described the information as interesting but not necessarily useful: “*I don’t know if it’s useful to me, but it’s interesting to see how many people did better after their surgery*” (15 F 70–79). Also, similar to Part 1, some participants said the EQ-5D-5L 1-year post-surgery information was not useful because they had already made their treatment decision:

*I already know I need surgery. This information won’t change my mind*” (12 M 70–79).

*I don’t want to have surgery and the doctors agreed with me*” (16 F 70–79).

### Visual appeal of versions for parts 1 and 2

Participants rated visual appeal as either a 5 (very visually appealing) or a 4 for the two versions of Part 1 (50% and 41.7%) and for the two versions of Part 2 (68% and 72%). For Part 1, 5 participants (20.8%) rated Version 2 as a 1 (not visually appealing). For Part 2, no participants (0%) rated either version as a 1(not visually appealing) (Table 3).

For both Parts 1 and 2, while a few participants commented that the prototypes “*looks adequate*” (14 M 50–59) or were “*visually clear*” (19 F 70–79), most recommended improvements for readability. Specifically, participants wanted *“Larger font size, larger pictures*” (08 F 60–69). Additionally, participants recommended increased colour contrast between items in Part 1: “*Make the colours more different to show they are five separate groups [levels]”* (21 M 60–69) or, *“White text on coloured backgrounds are hard to see so better to use a black text font”* (06 M 60–69). However, for Part 2, the contrast between white fonts on coloured textboxes was not as problematic with larger font in Version 1:. “*The larger lines make it easier to follow and read*” (12 M 70–79).

## Discussion

Overall, for Part 1, participants slightly preferred Version 1. For Part 2, participants preferred the visual appeal of Version 1, but the additional information of Version 2. Our intention in presenting two different versions for both Parts 1 and 2 was not to choose between them, but to gain insight into various elements included in the visualizations. Based on these results, we will use the preferred elements of both versions to finalize versions for pre-surgery and 1-year post-surgery (Table 1; columns b/c). For example, for both parts, we will increase comprehension and usefulness of the EQ-5D-5L pre-surgery and 1-year post-surgery information by explicitly describing the purpose of the information, the rationale for comparison to similar people, and what the information can be used for in supporting treatment decisions. We will improve the visuals for readability by increasing the size of fonts and images and enhancing contrast between elements for this patient population.

To our knowledge, this is the first study to elicit patient perspectives on visualizations specific to EQ-5D-5L data within a decision aid, including using the Sankey Diagram within a decision aid for clinical decision-making [20]. We believe the Sankey Diagram provides a better representation of individual change-over-time than the original presentation, which was not distinguishable from the cross-sectional presentation of pre-surgery status. Indeed, participants found it useful to know all of the possible outcomes of surgery, including the possibility of similar or worse outcomes. This implies that patients understood the presentation and interpreted the data correctly. Furthermore, our study demonstrates the application of recommendations and guidelines [8, 10] to promote patient understanding of PROMs data.

As noted previously, routine collection of PROMs is increasing within health systems. Incorporating historical PROMs data for *individualized* patient decision aids is a novel application at the *micro* level to enhance patients’ understanding of their options and risks and benefits to inform decision-making. While the potential benefit of the original version of the decision aid was demonstrated [5], improved visualization of the EQ-5D-5L data may further enhance its effectiveness. Our results on patients’ perspectives on the presentation of EQ-5D-5L data contribute to the knowledge on EQ-5D-5L applications within healthcare systems for clinical care. Our findings will be of interest to those involved in PROMs reporting, including the EuroQol community.

Next, we will conduct usability testing with patients for the updated online decision aid, including the EQ-5D-5L visualizations, using 3 validated instruments on usability, acceptability, and usefulness. Patients will self-administer the online decision aid to reflect routine use of the decision aid in clinical practice as intended. Following usability testing, the online decision aid will be finalized and scaled implementation will begin across all knee clinics in Alberta. Importantly, plans are underway at ABJHI to include EQ-5D-5L data from non-surgical patients to supplement the information presently available, providing a more comprehensive and holistic perspective for decisions for knee OA treatment, expanding EQ-5D-5L applications within healthcare systems for clinical care.

### Limitations

While our study has many strengths, there are some limitations. First, participants provided feedback on the EQ-5D-5L data without completing the entire decision aid. Furthermore, participants typically provided feedback on Part 1 or Part 2 separately, rather than sequentially, as will occur in the online decision aid. Removing this context may have influenced participants’ feedback. Second, for feasibility, we presented participants with paper copies of the prototypes while the decision aid will be online. To mimic an online presentation, we used landscape orientation. Regardless, this may have influenced participant feedback, particularly related to visual appeal. To minimize interfering with patient care, participants were recruited immediately after their screening appointment with a surgeon, at which time a treatment decision (surgical or non-surgical) may have been made. As such, participants may have underreported the usefulness of the EQ-5D-5L data presentations in decision-making. In contrast, in routine use the decision aid will be offered to patients prior to their screening appointment. With these limitations in mind, we elicited valuable participant feedback to improve the presentation of EQ-5D-5L data in the decision aid for comprehension, usability, and visual appeal.

## Conclusions

Based on the results of this study, we will make enhancements to the instructions and presentation of EQ-5D-5L data within the individualized decision aid to increase its usability for patients. These improvements, along with further usability testing of the entire individualized decision aid, will be made prior to large-scale implementation in routine clinical practice and may contribute to improved decision-making about TKA treatments. Further, our results on patients’ perspectives on the presentation of EQ-5D-5L data to support decision making for TKA contributes to the knowledge on EQ-5D-5L applications within healthcare systems for clinical care.

## Data Availability

Data are available from the corresponding author upon reasonable request and with permission of the University of Alberta.

## Abbreviations

ABJHI: Alberta Bone and Joint Health Institute
BMI: Body mass index
OA: Osteoarthritis
PROMs: Patient-reported outcome measures
SD: Standard deviation
TKA: Total knee arthroplasty
